# Beam wander relieved orbital angular momentum communication in turbulent atmosphere using Bessel beams

**DOI:** 10.1038/srep42276

**Published:** 2017-02-10

**Authors:** Yangsheng Yuan, Ting Lei, Zhaohui Li, Yangjin Li, Shecheng Gao, Zhenwei Xie, Xiaocong Yuan

**Affiliations:** 1 Nanophotonics Research Centre, Shenzhen University & key Laboratory of Optoelectronic Devices and Systems of Ministry of Education and Guangdong Province, College of Optoelectronic Engineering, Shenzhen University, Shenzhen, 518060, China; 2 Department of Physics, Anhui Normal University, Wuhu, 241000, China; 3 State Key Laboratory of Optoelectronic Materials and Technologies and School of Electronics and Information Technology, Sun Yat-sen University, Guangzhou, 510275, China; 4 Institute of Photonics Technology, Jinan University, Guangzhou, 510632, China

## Abstract

Optical beam wander is one of the most important issues for free-space optical (FSO) communication. We theoretically derive a beam wander model for Bessel beams propagating in turbulent atmosphere. The calculated beam wander of high order Bessel beams with different turbulence strengths are consistent with experimental measurements. Both theoretical and experimental results reveal that high order Bessel beams are less influenced by the turbulent atmosphere. We also demonstrate the Bessel beams based orbital angular momentum (OAM) multiplexing/demultiplexing in FSO communication with atmospheric turbulence. Under the same atmospheric turbulence condition, the bit error rates of transmitted signals carried by high order Bessel beams show smaller values and fluctuations, which indicates that the high order Bessel beams have an advantage of mitigating the beam wander in OAM multiplexing FSO communication.

Optical multiplexing has dramatically increased capacity and spectral efficiency in free space optical (FSO) communication and fiber optical communication. Multiplexing technologies have been proposed and demonstrated including spatial-division multiplexing^1^, polarization-division multiplexing^2^, frequency-division multiplexing^3^, wavelength-division multiplexing^4^ and mode-division multiplexing (MDM)^5^,^6^. Orbital angular momentum (OAM) multiplexing as a special case of MDM has been considered as a good candidate for high capacity optical communication. An optical beam carrying OAM, namely optical vortex (OV) beam, is associated with the helical shaped wavefront and the donut shaped intensity distribution. The phase term of exp(*inφ*) describes the helical transverse mode, where *n* is an integer indicating the topological charge of photons with 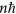 orbital angular momentum and *φ* is the azimuthal angle^7^. The OAM beams have widely been studied for particle trapping^8^, image processing^9^, astronomy^10^ and photon sorting^11^. The OAM beams with mutually orthogonal property have also been implemented for multiplexing/demultiplexing in FSO communication^5^,^6^.

In FSO communication, major challenges come from the atmospheric turbulence induced optical phase wavefront aberrations, intensity fluctuations and beam wanders^12^,^13^,^14^,^15^,^16^. The power fluctuations and mode crosstalk in optical communication system have been investigated theoretically and experimentally^17^,^18^,^19^,^20^,^21^,^22^. For OAM communication, the atmospheric turbulence influences for OAM states crosstalk have theoretically been studied in single-photon and multichannel communication system^18^,^19^. OAM multiplexing based outdoor light communication with the propagation distance ranging from 120 m to 3 km have also been demonstrated recently^20^,^21^,^22^. The high order Bessel beam with a field of *J*_*n*_(*k*_*r*_*r*) exp(*inφ*) can also be considered as a beam carrying a OAM state of exp(*inφ*), where *J*_*n*_ is the Bessel function of order *n, k*_*r*_ is the radial wave vector components, and (*r, φ*) are the polar coordinates^13^,^23^. The propagations of the Bessel beam and Laguerre Gaussian beam through turbulent atmosphere are comparably studied in ref. 24. The investigation results show, in the same condition, the variance of fluctuations of the Bessel beam are smaller than that of the Laguerre Gaussian beam with the same topological charge. Compared with the conventional OAM beams, the Bessel beams show advantages in non-diffraction and self-healing, which can benefit the beam propagation in turbulent atmosphere^24^,^25^,^26^. For the coherent and partially coherent Bessel beams propagating through turbulent atmosphere, the statistical properties such as variance of fluctuations of the OAM^24^, *M*^2^–factor^25^ and the variance of the displacement^26^ are theoretical studied. The results show the Bessel-Gaussian beams are less affected by the turbulent atmosphere with larger values of topological charges^24^,^25^,^26^. However, the ref. 26 provides only the numerical calculations rather than analytical derivations to predict the trends of the variance of the displacement. The Bessel beams with non-diffraction and self-healing properties have been used in optical and millimeter-wave communication^27^ and optical interconnect^28^,^29^. Therefore, the Bessel beams have the extraordinary properties may improve the OAM multiplexing based optical communications performances in turbulent atmosphere.

In this Letter, we investigate the high order Bessel beams based FSO communication with the turbulent atmosphere. Firstly, we derive an analytical formula for the beam wander of Bessel beams through turbulent atmosphere depending on the topological charge and turbulence strength. We also experimentally measure the beam wander of the Bessel beams with various topological charges. The results show that the Bessel beams with larger topological charges have smaller beam wander. Secondly, we build up a FSO communication system and characterize the Bessel beams based multiplexing technology in atmospheric turbulence. The measured BERs of the Bessel beams carrying the 10-Gbit/s on-off keying (OOK) signals can reach the forward error correction threshold.

## Results

### Principle and equations

**Figure 1:**
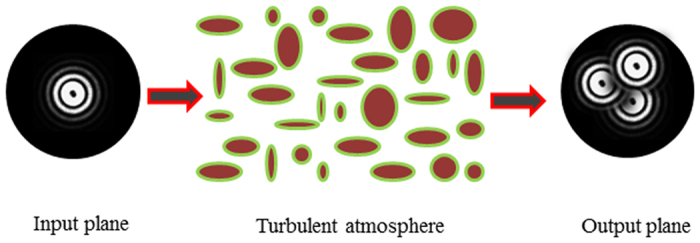
Schematic of the beam wanders for Bessel beam. Single Bessel beam in the input plane propagates through turbulent atmosphere. At the output plane, beam wanders are indicated by movements of the Bessel beam.

Figure 1 schematically shows the beam wander of Bessel beams propagating through turbulent atmosphere, corresponding to the movements of the instantaneous beam center at the output plane. Turbulent atmosphere induces the crosstalk among different OAM states. In the following sections, we will accomplish the theoretical model of beam wander for Bessel beam in turbulent atmosphere, experimentally characterize the beam wander of high order Bessel beams at two temperatures different from room temperature and demonstrate the FSO communication using multiple coaxial Bessel beams propagating in turbulent atmosphere.

The Bessel beams with infinite power do not exist in reality. The Bessel Gaussian beams (BGBs) can be considered as approximate Bessel beams in experiments. When the Gaussian beam incident on the holograph with phase function of exp(−*i*2*πr/r*_0_ + *inφ*), the *n*th order BGB can be generated^30^,^31^. The electric field of the *n*th order Bessel Gaussian beams is expressed as^25^


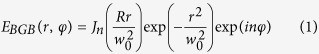


Here 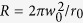, *w*_0_ is the waist width of the Gaussian beam.

Beam wander of the light propagating through the atmospheric turbulence can be described statistically by the variances of the displacements of the instantaneous beam center. The analytical expression of the beam wander available for weak, moderate and strong turbulence conditions is shown as^13^


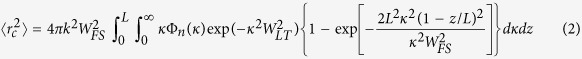


Here *κ* is the spatial frequency, *k* = 2*π/λ* is the wave number, *λ* is the wavelength, *L* is the propagation distance, and *z* is the distance of an intercept point on the propagation path. *W*_*LT*_ and *W*_*FS*_ are the long-term spot size with and without turbulence, respectively. Φ_*n*_(*κ*) is the spatial power spectrum of the refractive-index fluctuations.

We can use the geometrical optics approximation in Eq. (2) and simplify the last term as^13^


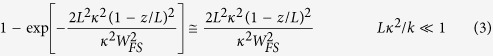


The Tatarskii spectrum is chosen as


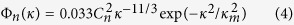


Here 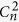 is the structure constant of the turbulent atmosphere, *κ*_*m*_ = 5.92/*l*_0_ with *l*_0_ as the inner scale.

By substituting Eqs (3) and (4) into Eq. (2), we obtain


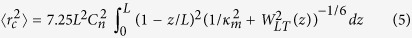


According to the result obtained in ref. 13, *W*_*LT*_ can be expressed as the mean squared beam width 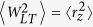.

Based on the second-order moments of the beam related to BGBs^25^, we obtain the long-term beam width of the BGBs propagating through atmospheric turbulence


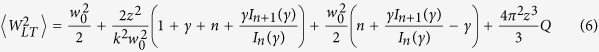


Here 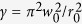, 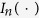 is the *n*th-order modified Bessel functions of the first kind, and 
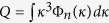
.

By substituting Eqs (4) and (6) into Eq. (5), the beam wander of the high order BGBs through turbulent atmosphere is obtained as Eq. (7).


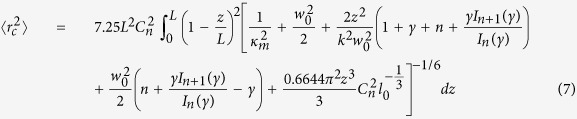


According to Eq. (5), the beam wander of Bessel Gaussian beams depends on the mean squared beam width. As shown in Eq. (6), there are larger mean squared beam widths with larger topological charges. From Eq. (7), the beam wander of the Bessel Gaussian beams propagation through turbulent atmosphere decreases with the increase of the topological charge.

### Experiment and results

**Figure 2:**
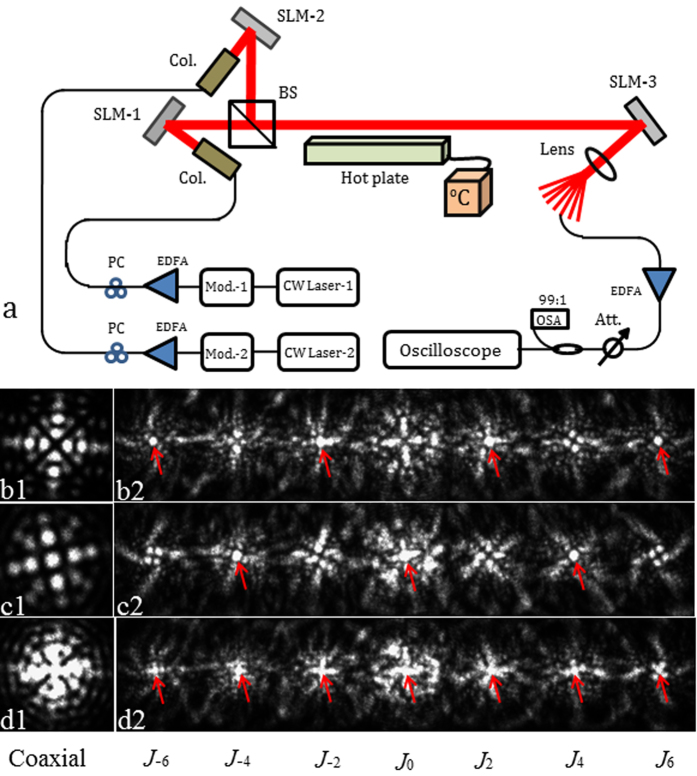
(**a**) Experimental setup of the Bessel beams through turbulent atmosphere in FSO communication system. (b1-d2) Images of the multiplexed/demultiplexed Bessel beams. (b1) the coaxial superposition Bessel beams with *J*_−6_, *J*_−2_, *J*_2_ and *J*_6_. (b2) the superposition Bessel beams (*J*_−6_, *J*_−2_, *J*_2_ and *J*_6_) demultiplexing simultaneously. (c1) The coaxial superposition Bessel beams with *J*_−4_, *J*_0_ and *J*_4_. (c2) the superposition Bessel beams (*J*_−4_, *J*_0_ and *J*_4_) demultiplexing simultaneously. (d1) the coaxial superposition Bessel beams with *J*_−6_, *J*_−4_, *J*_−2_, *J*_0_, *J*_2_, *J*_4_ and *J*_6_. (d2) The combined multiplexed beams (*J*_−6_, *J*_−4_, *J*_−2_, *J*_0_, *J*_2_, *J*_4_ and *J*_6_) demultiplexing simultaneously. The red arrows indicate light spots demultiplexed from Bessel beams. CW Laser: continuous wave laser; Mod.: Modulator; EDFA: erbium-doped fiber amplifier; PC: polarization controller; Col.: collimator; SLM: spatial light modulator; BS: beam splitter; Att.: attenuator; OSA: optical spectrum analyzer.

Figure 2(a) shows the experimental setup for Bessel beams based FSO communication in turbulent atmosphere. A continuous wave laser (CW Laser-1) at 1550 nm wavelength is fed into an optical modulator (Mod. −1) to produce the 10-Gbit/s OOK signals.

The signal laser is amplified by the high power erbium-doped fiber amplifier (EDFA), and then sent to the collimator (Col.) after the polarization controller (PC). In one branch, the collimated Gaussian beam with 4.65 mm diameter is projected onto reflective spatial light modulator (SLM-1) loaded with a special phase mask to generate the four collinear Bessel beams (Fig. 2b1). In the other branch, the collimated Gaussian beam is sent to the reflective SLM-2 loaded with another special phase mask to generate three coaxial superposition Bessel beams (Fig. 2c1). The combined multiplexed Bessel beams (Fig. 2d1) propagate through the turbulent atmosphere. The effects of atmospheric turbulence are simulated by the hot plate with controllable temperature. After propagating in turbulent atmosphere for 1.5 m, the collinear beams are sent to the reflective SLM-3 loaded with the optical vortex Dammann axicon grating.

The multiple high order Bessel beams generation with the Dammann grating have been demonstrated^32^,^33^. In this letter, the 1 × 7 optical vortex Dammann axicon grating is designed to detect the OAM states of the Bessel beams with topological charges of −6, −4, −2, 0, 2, 4 and 6. The grating parameters are chosen as follows: period *d* = 0.12 mm, topological charge interval *q* = 2 and the Bessel beam radius parameter *r*_0_ = 0.4 mm.

The demultiplexed beams focusing with the lens (focal length is 60 mm) are captured by the infrared camera or coupled into single mode fiber (SMF) for direct detection. Figure 2(b1–d2) shows the images of multiplex/demultiplex Bessel beams. Figure 2(b1) shows the superimposed Bessel beams of *J*_−6_, *J*_−2_, *J*_2_ and *J*_6_ generated by SLM-1, which are demultiplexed by the grating loaded on SLM-3 (Fig. 2(b2)). Figure 2(c1) shows the superimposed Bessel beams of *J*_−4_, *J*_0_ and *J*_4_ generated by SLM-2, which are demultiplexed by the grating loaded on SLM-3 (Fig. 2(c2)). Figure 2(d1) shows the superimposed Bessel beams of *J*_−6_, *J*_−4_, *J*_−2_, *J*_0_, *J*_2_, *J*_4_ and *J*_6_ combined by the beam splitter, which are demultiplexed by the grating loaded on SLM-3 (Fig. 2(d2)). The collected signal beams from the SMF is amplified by the low power EDFA. The signal is monitored by the optical spectrum analyzer (OSA). The received demultiplexed Bessel beams carrying signals input into the oscilloscope for the bit-error-rate (BER) measurement under various optical signal-to-noise ratios (SNRs) adjusted by the tunable power attenuator.

**Figure 3:**
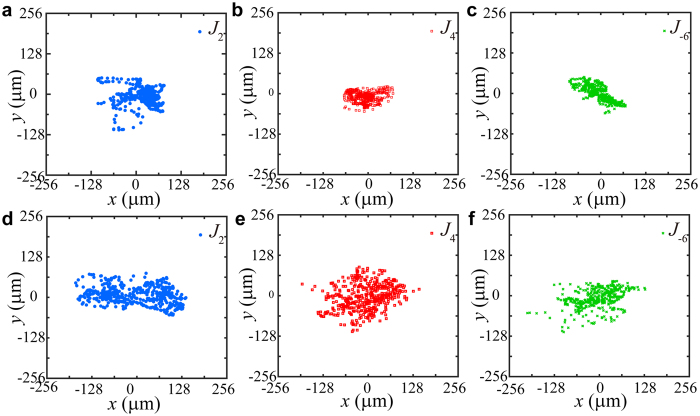
Displacements of high order Bessel beams (*J*_2_, *J*_4_ and *J*_−6_) after 1.5 m propagations in turbulent atmosphere at different temperatures. Each dot indicates the central position of the Bessel beams extracted from 488 imaging pictures captured during 30 seconds. The temperature difference from room temperature (T = 300 K) is δT = 23 K in (**a**–**c**). δT = 43 K in (**d**–**f**).

Figure 3 shows central positions of high order Bessel beams (*J*_2_, *J*_4_ and *J*_−6_) in turbulent atmosphere at the receive plane at 1.5 m away. For each figure, the superimposed dots are the centers of the Bessel beams recorded by the IR camera in 30 seconds with 448 frames.

The atmospheric turbulence is emulated by the hot plate and the turbulence strength is controlled by temperature difference δT^16^. The temperature gradient will generate convection air flow induced vertical turbulent atmosphere. In order to avoid air vortices, some iron needles are placed on the hot plate to form the inhomogeneous underlying surface. The beam wander can be extracted according to the displacements of the beams propagating through turbulent atmosphere. The structure constant 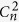 can be calculated by the standard deviation of the displacement^13^,^20^. As shown in Fig. 3, the displacements of the Bessel beams increase with the temperature difference increasing for the same topological charge value. The displacements of the Bessel beams increase with the topological charge decreasing, for a fixed temperature difference.

**Figure 4:**
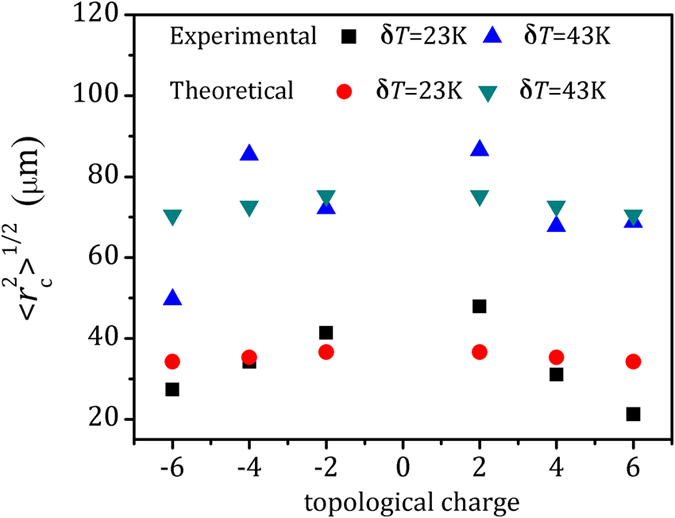
Beam wanders of the Bessel beams (*J*_2_, *J*_4_ and *J*_−6_) from theoretical calculation and experimental results at two temperatures difference from room temperature δT = 23 K and δT = 43.

As shown in Fig. 4, the beam wanders of the Bessel beam from the theoretical calculations and experimental results show the same trend. The beam wanders are obtained from mass derivations of the Bessel beam displacements experimentally. As a comparison, we also theoretically calculate the beam wanders of Bessel beams with the same topological charges as demonstrated in the experiments using Eq. (7). The beam wander values 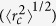 of the Bessel beams are 27.4 μm, 34.2 μm, 41.4 μm, 47.9 μm, 31.1 μm, 21.3 μm with δT = 23 K, and 49.7 μm, 85.4 μm, 72.1 μm, 86.5 μm, 67.8 μm, 68.8 μm with δT = 43 K for *J*_−6_, *J*_−4_, *J*_−2_, *J*_0_, *J*_2_, *J*_4_ and *J*_6_. The Bessel beams shows smaller beam wanders with lower temperature difference and larger topological charge. Turbulence as a random process cannot be simply extracted from the emulation environment. The ensemble average is used to describe the random field. The structure constant 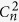 is the parameter to indicate the strength of the turbulence, which is typically calculated by experimental data. The experimental results are based on time averages^13^,^16^. Using the beam wanders values, the structure constant of the turbulent atmosphere are calculated as 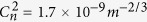 with δT = 23 K and 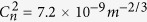 with δT = 43 K. We attribute the differences between the theory and the experiment to the statistic of the data in atmospheric turbulence.

**Figure 5:**
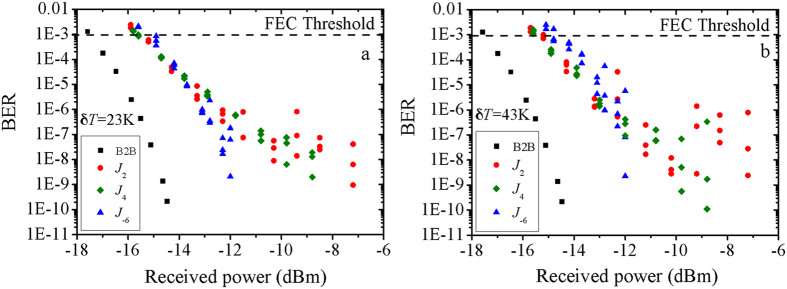
The BER curves of *J*_2_, *J*_4_ and *J*_−6_ for coaxial Bessel beams 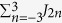 propagate through turbulent atmosphere demultiplexing in different temperature difference (**a**) δT = 23 K and (**b**) 43 K.

Figure 5 shows the BER measurements from the experimental setup as described above. The signal transmitted by the two branched can be considered as unrelated. Therefore signal carried by any Bessel beams from the other branch contribute to crosstalk in the communication system. According to our previous work^5^, the adjacent channels contribute the most portion of the crosstalk. The unrelated signals from two paths can satisfy the BER measurement request for multiple channels^22^,^34^. In our experiment, the SNR is adjusted by an attenuator after the turbulent atmosphere. In principle the beam wander value is irrelevant to power at the fixed turbulence strength^13^. Figure 5(a) shows the BER of the Bessel beams *J*_2_, *J*_4_ and *J*_−6_ with the temperature difference from room temperature δT = 23 K. There are BER fluctuations due to beam wanders of Bessel beam in atmospheric turbulence. Since the turbulent atmosphere is random varying during each measurement, the BER values recorded show differences. Especially for the relative low BER value, the random effect is more obvious. With this fixed temperature difference, the Bessel beams with larger topological charges show better communication performance in terms of BER fluctuations and values, which is consistent with the theoretical and experimental results of the beam wanders as shown above. Further increasing the temperature difference to δT = 43 K, the BER measurements still follow the same trend. Although the turbulent atmosphere induce beam wanders and BER fluctuations, all the BERs of the coaxial Bessel beams are still below the forward error correction (FEC) threshold of 1 × 10^−3^ in this FSO communication link.

**Figure 6:**
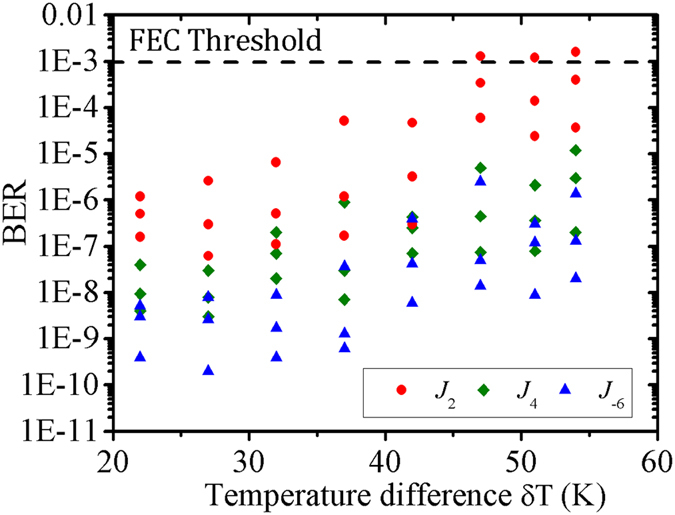
The BER of *J*_2_, *J*_4_ and *J*_−6_ with temperature difference δT at fixed received power of −12 dBm.

Figure 6 shows the BER values varying according to temperature difference δT. The received power is fixed at −12 dBm and the values of the BER are recorded in 30 seconds. In the same condition, the BER shows relative smaller values with larger topological charge. This result is consistent with our theoretical models for the beam wander of the Bessel beams in turbulent atmosphere.

## Discussion

In summary, the beam wanders of the Bessel beams propagating through turbulent atmosphere have been theoretically and experimentally studied. Both experimental data and theoretical calculations show the same trend, that the beams with large topological charge value suffer less effects of turbulent atmosphere. We also multiplex/demultiplex collinear OAM states of Bessel beams by an optical vortex Dammann axicon grating in an FSO link, where the atmospheric turbulence is emulated by a hot plate. The Bessel beams relieved the beam wanders and satisfied the BER criterion for standard OAM multiplexing/demultiplexing in FSO system.

## Methods

A standard Gaussian beam carrying high speed signals shines on the phase-only SLM to generate the multiple OAM states of Bessel beams in one branch. The topological charge interval of the Bessel beams is 4. In the other branch multiple Bessel beams carried different signals with the topological charge interval of 4. The two branches multiple Bessel beams are coupled by the beam splitter to generate the multiple OAM states of Bessel beams with the topological charge interval of 2. The perturbation of the turbulent atmosphere is employed by the hot plate. The collinear OAM states of Bessel beams propagating through turbulent atmosphere are simultaneous demultiplexed by the SLM loaded by the optical vortex Dammann axicon grating. The SLM has a resolution of 1920 × 1080 pixel and the pixel pitch size is 8 μm.

## Additional Information

**How to cite this article**: Yuan, Y. *et al*. Beam wander relieved orbital angular momentum communication in turbulent atmosphere using Bessel beams. *Sci. Rep.* **7**, 42276; doi: 10.1038/srep42276 (2017).

**Publisher's note:** Springer Nature remains neutral with regard to jurisdictional claims in published maps and institutional affiliations.
